# PD-1, PD-L1 (B7-H1) and Tumor-Site Immune Modulation Therapy: The Historical Perspective

**DOI:** 10.1186/s13045-017-0403-5

**Published:** 2017-01-25

**Authors:** Jun Wang, Ruirong Yuan, Wenru Song, Jingwei Sun, Delong Liu, Zihai Li

**Affiliations:** 1 Yale University School of Medicine, New Haven, CT 06510 USA; 2 Veterans Health Administration Medical Center, East Orange, NJ 07018 USA; 3 The Chinese American Hematologist and Oncologist Network (CAHON), Scarsdale, NY 11577 USA; 4 New York Medical College, Valhalla, NY 10595 USA; 5 Medical University of South Carolina, Charleston, SC 29425 USA

**Keywords:** B7-H1, PD-L1, PD-1, CTLA-4, CD28, immune checkpoint, immunotherapy, immuno-oncology, T cells, tumor-site immune modulation therapy

## Abstract

The current success of targeted inhibition against cytotoxic T-lymphocyte-associated protein 4 (CTLA-4) and Programmed Death 1/Programmed Death Ligand 1 (PD-1/PD-L1, herein collectively referred to as PD) pathways is hailed as a cancer immunotherapy breakthrough. PD-L1, known also as B7 homolog 1 (B7-H1), was initially discovered by Dr. Lieping Chen in 1999. To recognize the seminal contributions by Chen to the development of PD-directed therapy against cancer, the Chinese American Hematologist and Oncologist Network (CAHON) decided to honor him with its inaugural Lifetime Achievement Award in Hematology and Oncology at the CAHON’s 2015 annual meeting. This essay chronicles the important discoveries made by Chen in the exciting field of immuno-oncology, which goes beyond his original fateful finding. It also argues that PD-directed therapy should be appropriately considered as *Tumor*-*Site Immune Modulation Therapy* to distinguish it from CTLA-4-based immune checkpoint blocking agents.

## Background

Monoclonal antibodies targeting the PD pathway have become a critical breakthrough in our long fight against cancer [1, 2]. Distinct from any previous anti-cancer drugs, PD-based cancer therapy neither directly targets tumors, nor simply revamps the immune system non-specifically. It depends on the strategic modulation of a key tumor immune evasive mechanism featured by the PD-L1 (B7-H1) molecule, and controls tumor growth by resetting immune responses in the tumor microenvironment to the homeostatic and beneficial level [3, 4]. Currently, several anti-PD therapeutic agents have been approved for the treatment of multiple cancer types including lung cancer, head and neck cancer, melanoma and others in the United States, Europe, as well as in Japan and other parts of the world. Numerous clinical trials are ongoing worldwide in order to broaden and increase the utility of anti-PD therapeutics to most if not all human cancers, thanks to the impressive clinical efficacy with favorable toxicities of these novel agents. The successful development of PD-modulating medicines has revolutionized the field of immuno-oncology in an unprecedented way, and opens the door for *Tumor*-*Site Immune Modulation Therapy* [5] that will profoundly impact basic and clinical immune-oncology research. Indeed, many outstanding reviews have been written on this topic [3–12]. Nevertheless, in this essay, we review the key milestones in the history of anti-PD drug development, and specifically highlight some of Lieping Chen’s contributions to this revolutionary cancer treatment modality.

### History of anti-PD drug development and the roles of Lieping Chen

The success of anti-PD drug development benefited from the advancement of our fundamental understanding of both cancer biology and the immune system. Cancer originates from the mutated self of the host in the setting of genotoxic insults with molecular hallmarks of genetic instability and heterogeneity [13, 14]. Most conventional therapies, including radiation, chemotherapy and even molecular-targeted therapies, directly target cancers themselves with improving, but still less desirable efficacy due to significant side-effects, drug resistance and recurrence of more aggressive malignant clones [8, 13, 15]. The limitations of conventional therapies are especially evident for late stage and advanced cancers. In contrast, the immune system, especially the adaptive arm of immunity such as T cells, is capable of surveilling against mutated antigens in tumor cells and controlling tumor growth through specific immune effector mechanisms. Importantly, these immune cells evolve in parallel with tumors, and are capable of sustainable tumor recognition and killing [16, 17]. However, surveillance efficacy often fails due to multiple tumor immune evasive mechanisms, resulting in the outgrowth and metastasis of cancer cells [18, 19]. Efforts in understanding the basic mechanisms of immune tolerance in general and the primary immune evasive mechanisms by cancer cells in particular have been the key to the successful development of drugs targeting the PD-1/PD-L1 pathway.

**Fig. 1:**
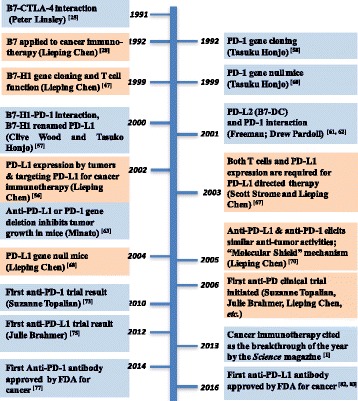
Timeline for major events leading to the development of anti-PD drugs. The contributions by Lieping Chen are highlighted in light orange. The contributions by Lieping Chen are highlighted in light orange

Many individuals contributed to the success of PD-directed cancer therapy (Table 1 and Fig. 1) [2]. Among them, Lieping Chen’s work is the focus of this review. In the early span of the 1990s, Chen was convinced that in the tumor microenvironment, there exist specific molecular pathways that are primarily responsible for immune evasion, and that this concept could be harnessed for more effective cancer therapy. His passion has been devoted towards the search for these elusive molecules ever since. Trained as a physician-scientist in China, Chen quickly became convinced that basic medical research holds the key to cancer cure. He earned his M.S. degree at the Cancer Institute of Peking Union Medical College in 1986 before obtaining his Ph.D. from Drexel University in 1989. He then undertook his postdoctoral training at the University of Washington, Seattle in 1990, all along focusing on immunology. During that period, important discoveries were made regarding the major immune cell populations with different immune functions, distinguished by cell surface molecules, including CD3, CD4, CD8, T cell receptor (TCR) and others [20–23]. More importantly, the guiding principle of T cell activation and tolerance began to emerge [21]. Among those important events, the discovery of CD28, CTLA-4 and their ligands B7 (B7.1 and B7.2) was the key development in the field [24, 25] which vindicated the two-signal hypothesis for lymphocyte activation proposed by Bretscher and Cohn [26], and extended by Lafferty and his colleagues [27]. The first signal is via TCR triggered by an MHC-antigenic peptide complex, whereas the second signal, also known as a co-stimulation signal, is provided by co-stimulatory molecules expressed on the surface of antigen presenting cells (APC) and T cells (like B7-CD28 pathway). Based on this principle, Chen and his colleagues, for the first time, demonstrated that stably-enforced expression of B7 molecules in cancer cells elicited strong anti-tumor immune responses, leading to eradication of distal tumors [28]. This effect is mainly CD28 dependent: upon ligation of the same B7 ligand, CD28 delivers an essential signal for naïve T cell activation [29], which is in contrast to the CTLA-4 molecule behaving as a T cell “checkpoint” receptor with inhibitory activities [30–33]. Blocking CTLA-4 for immunotherapy of cancer was later steered by James Allison who has played crucial roles in the renaissance of cancer immunology [34–37], whereas Chen’s earlier work laid the groundwork for the therapeutic potential of manipulating co-stimulatory molecules against cancer. However, the ectopic expression of costimulatory molecules like B7 on tumor cells worked effectively against multiple murine tumor models, its application is currently limited in cancer patients [38]. Additionally, since both B7 ligands and CD28/CTLA-4 receptors are expressed broadly without tumor specificity and that they play essential roles in the control of general immune homeostasis, targeting this pathway could be met with severe autoimmune toxicities, which was shown to be the case in the clinical trials of TGN1412, an anti-CD28 super agonist antibody [39], and the anti-CTLA-4 antibodies, ipilimumab and tremelimumab [40–42]. Chen was determined to uncover *tumor*-*specific* immune evasion mechanisms and find ways to block them.

**Table 1:** Major contributions to the development of targeted cancer immunotherapeutics against CTLA-4 and PD-1/PD-L1 Pathways ^a^

Contributions	CTLA-4	PD-1	PD-L1 (B7-H1)
Gene Cloning	Pierre Goldstein (1987) [59]	Tasuku Honjo (1992) [57]	**Lieping Chen (1999)** [47]
Inhibitory Function	Jeffery Bluestone (1994) [30] Arlene Sharpe (1995) [32] Tak Mak (1995) [33]	Tasuku Honjo (1999) [60]	**Lieping Chen (1999)** [47] Tasuku Honjo, Clive Wood (2000) [57] ^b^ **Lieping Chen (2004)** [68]
Ligand-receptor Interaction	Peter Linsley (1991) [25]	Tasuku Honjo, Clive Wood (2000) [57] ^b^	Tasuku Honjo, Clive Wood (2000) [57] ^b^
Function in cancer immunity	James Allison (1996) [34]	NagahiroMinato (2002) [63] **Lieping Chen (2005)** [70] Tasuku Honjo (2005) [71]	**Lieping Chen (2002)** [56] Nagahiro Minato (2002) [63]

^a^ The discovery of PD-L2 was made by Gordon Freeman and Arlene Sharpe (2001)[61], and Drew Pardoll (2001)[62]. Subsequent work on PD-L2 is not highlighted here

^b^ Gordon Freeman, Andrew Long and Yoshiko Iwai are the three co-first authors of this work, which renamed B7-H1 (its gene cloned one year earlier) to PD-L1

During his seven-year tenure at Bristol-Myers Squibb (1990–1997), Lieping Chen evaluated the immune functions and potential anti-tumor effects of many cell surface molecules on T cells, especially 4-1BB (CD137), a molecule specifically expressed by activated T cells and serving as another co-stimulatory receptor. The Chen group were the first to reveal the potent anti-tumor effect of the anti-4-1BB agonistic antibody in both immunogenic and non-immunogenic tumor models [43], making 4-1BB an attractive target for immuno-oncology [44]. Some promising results from the anti-4-1BB clinical trials were recently reported [45]. Although the expression of 4-1BB ligand is relatively broad, 4-1BB expression could accurately identify tumor-reactive T cells [46]. Even so, Chen believed that immune-regulatory molecules with higher tumor specificity remained to be identified. He returned to academia and joined the Mayo Clinic to continue searching for these pathways. Inspired by the progress of the Human Genome Project, Chen turned to informatics to discover candidate molecules from the human expressed sequence tag (EST) libraries based on their predicted homology to B7 family molecules. This effort was incredibly successful; he made several seminal discoveries of a series of novel B7 family members, including B7-H1 (PD-L1) [47], B7-H2 [48], B7-H3 [49], B7-H4 [50], B7-H5 [51], PD-1H (VISTA) [52] and so on [53–55]. In a series of papers, Chen and his colleagues completed the fundamental characterization of the B7-H1’s biological function and provided the very first proof of anti-tumor effects via blockade of B7-H1 [47, 56], which mainly serve as a ligand to PD-1 receptor on T cells [57], a molecule discovered earlier by Tasuku Honjo in Japan [58]. In 2004, Chen joined the Johns Hopkins University School of Medicine and collaborated with Suzanne Topalian, Julie Brahmer and other clinical investigators to initiate the first clinical trial of anti-PD-1/PD-L1 pathway therapies in patients with cancer. It was this clinical study that opened the floodgate of PD-1/PD-L1 pathway-directed cancer immunotherapy.

The key events that led to the successful development of anti-CTLA-4 and anti-PD-1/PD-L1 drugs are chronicled below (Fig. 1 and Table 1):

- From 1990 to 1991, Peter Linsley from the Bristol-Myers Squibb discovered the interaction between CD28, CTLA-4, and B7 [24, 25]. The B7 pathway was later generally considered as an essential co-stimulatory molecule for naïve T cell activation [21, 54].
- In 1992, by stably expressing B7 molecule in the tumor cells, Lieping Chen provided the theoretical basis for the therapeutic potential of manipulating the expression of co-stimulatory molecules in the tumor microenvironment for cancer immunotherapy [28, 29].
- In 1992, Tasuku Honjo cloned the PD-1 gene (*Pdcd1*) from immune cell lines undergoing apoptosis [58].
- In 1994, Jeffery Bluestone first identified the inhibitory function of CTLA-4, and also categorized CTLA-4 as the first cell surface T cell inhibitory receptor [30]*.
  *Note: During the process of drug development targeting this molecule, Pierre Goldstein cloned CTLA-4 gene (*Ctla4*) [59] and Peter Linsley discovered the receptor-ligand interaction between B7 and CTLA-4 [25] (Table 1). Arlene Sharpe and Tak Mak subsequently reported the fatal autoimmune diseases of *Ctla4*-deficient mice [32, 33]. James Allison characterized the anti-tumor effect of antibody targeting CTLA-4 [34]. The anti-CTLA-4 antibody, ipilimumab, was later approved by the U.S. Food and Drug Administration (FDA) for treatment of melanoma in 2011 [37].
- In 1997, Lieping Chen identified the potent anti-tumor effect of agonistic antibody targeting 4-1BB, another co-stimulatory receptor on T cells, which further inspired the field of cancer immunotherapy [43].
- Around 1997, given the progress of the Human Genome Project, Lieping Chen’s group started to search for B7-like molecules from the human EST libraries, thus began his seminal works on expanding the members of the B7 family.
- In 1999, Chen cloned the first B7 homolog, the human B7-H1 gene, and identified its inhibitory activity on T cells by inducing IL-10 [47]. During the following years, Chen’s group also cloned B7-H2 [48], B7-H3 [49], B7-H4 [50], B7-H5 [51] and PD-1H [52].
- In 1999, Tasuku Honjo discovered that the PD-1 gene (*Pdcd1*) knockout mice have mild autoimmune symptoms, which revealed the inhibitory function of PD-1 in preventing autoimmunity [60].
- In 2000, Tasuku Honjo and Clive Wood discovered the interaction between B7-H1 and PD-1, and changed the name of B7-H1 to PD-L1 [57].
- In 2001, Arlene Sharpe and Gordon Freeman discovered another PD-1 ligand, PD-L2 (Programmed Death Ligand 2), which also shows inhibitory activity to T cells [61]. Drew Pardoll’s group identified PD-L2 around the same time, and named this molecule B7-DC for its specific expression on dendritic cells [62].
- In 2002, Lieping Chen discovered the critical role of B7-H1 (PD-L1) as a potential immune evasion mechanism in the tumor microenvironment. B7-H1 is found to be overexpressed in many human tumor tissues, but minimally detected in the normal tissues, which was mainly regulated by IFN-γ [56]. Most importantly, antibody-targeting B7-H1 could restore T cell function and control tumor growth both *in vitro* and *in vivo* [56]. Subsequent works by Nagahiro Minato [63] and Weiping Zou [64] further supported this finding. Moreover, Chen’s study suggested the existence of other receptor(s) for B7-H1, which was later validated by a follow-up mutation study made by Chen [65], and finally led to the discovery of B7-1 as another B7-H1 inhibitory receptor by Arlene Sharpe and Gordon Freeman [66].
- In 2003, Scott Strome and Lieping Chen showed that B7-H1 overexpression in tumor cells and T cell activation are two indispensable pre-conditions for the potent anti-cancer effect of antibodies blocking this pathway [67].
- In 2004, Lieping Chen discovered that the B7-H1 gene (*Cd274*) null mice have some spontaneous accumulation of activated CD8^+^ T cells in the liver, but do not have overt autoimmune manifestations. This work further proved the inhibitory function of B7-H1 and predicted the acceptable safety profile of B7-H1-targeted therapy [68]. An independent study by Arlene Sharpe and Gordon Freeman using *Cd274*-deficient mice also proved that PD-L1 negatively regulates T cells [69].
- In 2004, Lieping Chen joined the Johns Hopkins University School of Medicine, and contributed to the development of the first-in-human trial concept on antibodies targeting the PD-1/PD-L1 pathway for the treatment of advanced cancers.
- In 2005, Lieping Chen demonstrated that antibodies blocking either B7-H1 or PD-1 could promote antitumor immune responses, and proposed the “Molecular Shield” mechanism of PD-L1 on tumors that offers resistance to cytotoxic T lymphocytes (CTL) [70]. Tasuku Honjo also demonstrated that PD-1 blockade by genetic manipulation or antibody treatment inhibited hematogenous spreading of tumor cells [71].
- In 2006, Rafi Ahmed characterized a role of the PD-1/PD-L1 pathway in T cell exhaustion with the lymphocytic choriomeningitis virus (LCMV) chronic infection model [72].
- In 2006, the first human cancer clinical trial targeting the PD-1/PD-L1 pathway was launched in the Johns Hopkins Hospital.
- In 2010, the first clinical observation on anti-PD-1 treatment was reported by Suzanne Topalian [73].
- In 2012, the results of the first two large anti-PD-1 and anti-PD-L1 clinical trials in the Johns Hopkins Hospital, the Yale-New Haven Hospital and others were reported [74, 75].
- In 2006, Lieping Chen’s group developed a sensitive and effective immunohistochemistry staining protocol for detecting PD-L1 expression in cancer cells, and pointed out the value of PD-L1 staining in tumor sections on the prediction of anti-PD-1/PD-L1 clinical efficacy in 2012. Chen also refined his theory on anti-PD-1/PD-L1 therapy by proposing the adaptive resistance concept [76].
- In 2013, cancer immunotherapy was selected as the breakthrough of the year by the *Science* magazine [1, 2].
- In 2014, anti-PD-1 antibodies (nivolumab and pembrolizumab) were approved in the United States and Japan for treatment of advanced metastatic melanoma [77], and subsequently for treatment of many other cancer types in 2015–2016 [78–82].
- In 2016, the anti-PD-L1 antibody, atezolizumab, was approved by the FDA for the treatment of advanced urothelial carcinoma and non-small cell lung cancer [82, 83].

### Anti-PD modality and tumor-site immune modulation therapy

The timeline in the history of anti-PD drug development (Fig. 1) spans from the understanding of the B7 pathway in regulating T cell responses to the discovery of the PD pathway with tumor-site immune modulation properties, reflecting our increased understanding of both T cell biology and tumor immunity. This advancement resulted from the progress of research in the field of both immunology and oncology, eventually leading to the birth of immuno-oncology. Ironically, anti-PD antibodies did not show clear anti-tumor effects in many animal models in the early days of research, raising skepticism among many over their therapeutic application. However, Lieping Chen and his colleagues proposed that the potency of anti-PD therapy depends on both the existence of immune cells especially T cells in the tumor site, as well as PD-L1 expression by the tumor cells [67, 70, 84]. He steadfastly and passionately championed the clinical development of agents targeting this pathway. Thus, Chen played key roles in advancing the anti-PD drug program in the areas of basic research as well as in clinical translation (Table 1), for which he was recognized with the 2014 William Coley Award in Basic and Tumor Immunology [85] (shared with Tasuku Honjo, Arlene Sharpe and Gordon Freeman), the 2015 Lifetime Achievement Award in Hematology and Oncology by CAHON (www.cahon.org, and this paper), and the 2016 American Association of Immunologists (AAI)-Steinman Award for Human Immunology Research (http://www.aai.org/Awards/Archive/index.html).

**Fig. 2:**
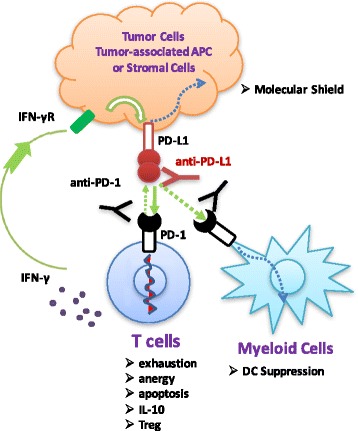
Mechanism of the PD pathway in driving tumor-associated immune evasion. Tumor cells, tumor-associated antigen-presenting cells (APCs), and stromal cells upregulate PD-L1 in response to ongoing immune responses, mainly through the action of IFN-γ. The ligation of PD-1 by PD-L1 delivers inhibitory signals to T cells, leading to T cell anergy, functional exhaustion, and apoptosis. PD-1-PD-L1 interaction also favors conversion of T cells to the regulatory T cell (Treg) phenotype with secretion of inhibitory cytokines, such as IL-10. PD-1 on myeloid cells also impairs dendritic cell functions. In addition, PD-L1 reversed signaling on tumor cells can serve as a “Molecular Shield” protecting tumor cells from CTL-mediated killing. IFN-γR: IFN-γ receptor

**Fig. 3:**
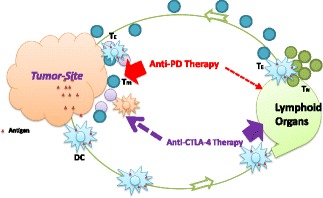
Anti-PD modality: *Tumor*-*site immune modulation therapy*. Anti-PD therapy is mechanistically distinct from anti-CTLA-4 therapy: the latter affects immune responses more systemically, whereas anti-PD therapy primarily targets its actions at the tumor site. Anti-PD modality is thus capable of repairing tumor-induced immune defects, ultimately leading to resetting of the anti-tumor immunity to a desirable level. T_N_: Naïve T cells; T_E_: T effector cells; T_m_: memory T cells; DC: dendritic cells

Targeting the PD pathway for treatment of cancer is unique in several aspects, including the following (Figs. 2 and 3):

First, the action of anti-PD therapy is primarily localized to the tumor site. The ligand for PD-1, PD-L1 (B7-H1), has high expression on tumor cells. It is absent in the majority of normal tissues, but could be induced in the tumor microenvironment by ongoing immune responses, mostly by the cytokine IFN-γ [3, 56]. This tumor-localized effect of PD-L1 dictates that the antibodies targeting either ligand or receptor will work in the tumor microenvironment with ongoing immune responses. In contrast, PD-L2, another ligand of PD-1, has weak expression in tumors and is constitutively expressed by dendritic cells [53, 54]. The expression pattern of PD-L2 likely explains why targeting PD-L2 has only minor anti-tumor effects, although PD-L2 and PD-L1 have similar binding affinity to PD-1, and their inhibitory effects on T cells *in vitro* are comparable.

Second, targeting the PD pathway repairs or resets tumor-associated immune defects. The PD-L1 molecule is a key mechanism for tumor-mediated immune evasion [3]. The ligation of PD-L1 to PD-1 causes functional defects in T cells through several different mechanisms (Fig. 2), including anergy to antigen stimulation [86–88], functional exhaustion [72], apoptosis [56], induction of immune suppressor cells [89, 90], and secretion of inhibitory cytokines, such as IL-10 [47]. PD-1 on myeloid cells also impairs dendritic cell functions [91]. In addition, PD-L1 reverse-signaling on tumor cells was found to serve as a “Molecular Shield,” protecting the tumor from killing by CTLs [70, 92]. Importantly, this type of immune defect is not permanent, and can be restored by termination of this pathway, especially by the antibody blockade of either PD-L1 or PD-1 [70]. Moreover, anti-PD therapy may reset the global anti-tumor immune defects possibly by yet unknown “cascade reaction” mechanisms, as profound and sustainable therapeutic effects have been observed in many patients receiving this therapy [3].

Third, the PD blockade aims to normalize the anti-tumor immune response but not over-exuberate immune responses in general. Blockade of either PD-L1 or PD-1 is not to simply amplify immunity, but to re-adjust the anti-tumor immune responses to a desirable level (Fig. 3). Given the mild and rare autoimmune symptoms observed in PD-L1 and PD-1 gene-deficient mice [60, 68] and weak expression of these molecules by normal tissues [56, 93], targeting the PD pathway in the tumor settings will normalize anti-tumor immunity but spare the normal peripheral tolerance mechanism against self-antigens [3]. Thus, anti-PD treatment has an understandably great safety window [94].

These abovementioned features of the PD pathway are very unique among the many pathways currently tested for cancer immunotherapy. Strictly speaking, PD-1/PD-L1 is the only known pathway responsible for key *tumor*-*specific* immune evasion mechanisms so far. Yet, both PD-1/PD-L1 and CTLA-4 are often lumped together as “immune checkpoints” [6, 8, 31, 95], which is an immunological term defining a plethora of inhibitory pathways in the control of physiological immune responses [6]. This concept does not distinguish the immune modulating role of anti-PD therapy from the actions of anti-CTLA-4 therapy [3, 7] (Fig. 3). An alternative term of “*Tumor*-*Site Immune Modulation Therapy*” might better describe the attributes of anti-PD therapy [5], which is characterized by targeting molecules responsible for tumor-site specific immune evasion mechanisms, resetting and restoring the anti-tumor immunity back to a desirable level. Such action is clearly different from CTLA-4 blockade, which overdrives systemic T cell immunity including self-reactive T cell responses.

Lieping Chen continues his journey to discover more PD-like molecules, aiming to convert human cancers to chronic and manageable diseases via exploiting the power of the hardwired immune system. His contributions to this field have cemented his place in immuno-oncology.
